# Sodium–glucose cotransporter 2 inhibitors in breast cancer patients on endocrine therapy: a targeted strategy to mitigate long-term cardiometabolic risk

**DOI:** 10.1186/s12967-026-08473-8

**Published:** 2026-06-22

**Authors:** Vincenzo Quagliariello, Massimiliano Berretta, Irma Bisceglia, Matteo Barbato, Raffaele Arianna, Pietro Forte, Jacopo Santagata, Carlo Maurea, Maria Laura Canale, Andrea Paccone, Alessandro Inno, Cristiana D’Ambrosio, Stefano Oliva, Christian Cadeddu Dessalvi, Tiziana Di Matola, Domenico Gabrielli, Nicola Maurea

**Affiliations:** 1 Division of Cardiology, Istituto Nazionale Tumori- IRCCS- Fondazione G. Pascale, Napoli, Italy; 2 Department of Clinical and Experimental Medicine, University of Messina, Messina, Italy; 3 Servizi Cardiologici Integrati, Dipartimento Cardio-Toraco-Vascolare, Azienda Ospedaliera San Camillo Forlanini, Rome, Italy; 4 Neurology Division, Azienda Ospedaliera di Rilievo Nazionale Antonio Cardarelli, Naples, Italy; 5 U.O.C. Cardiologia, Ospedale Versilia, Lido di Camaiore, LU Italy; 6 Medical Oncology, IRCCS Ospedale Sacro Cuore Don Calabria, Negrar di Valpolicella, VR Italy; 7 Division of Cardiology, P.O. “F. Veneziale”, Isernia, Italy; 8 Cardio-Oncology Unit, IRCCS Istituto Tumori, “Giovanni Paolo II”, 70124 Bari, Italy; 9 Department of Medical Sciences and Public Health, University of Cagliari, Cagliari, Italy; 10 Azienda Ospedaliera Dei Colli, Naples, Italy; 11 U.O.C. Cardiologia, Dipartimento Cardio-Toraco-Vascolare, Azienda Ospedaliera San Camillo Forlanini, Rome, Italy; 12 Fondazione per il Tuo cuore - Heart Care Foundation, Firenze, Italy

**Keywords:** Cardio-oncology, Cardiotoxicity, Gliflozins, SGLT2, Cardio-renal, Inflammation

## Abstract

**Background:**

Hormone receptor–positive breast cancer (HR+ BC) represents the most common breast cancer subtype and is increasingly characterized by long-term survivorship complicated by substantial cardiometabolic and cardiovascular morbidity. Endocrine therapies, including aromatase inhibitors, selective estrogen receptor modulators and degraders, and ovarian suppression, remain central to disease control but promote metabolic dysfunction, vascular injury, and systemic inflammation, thereby increasing cardiovascular disease (CVD) risk and potentially contributing to endocrine resistance. These alterations are also associated with adverse changes in adipose tissue distribution, increased visceral fat accumulation, and dysregulated lipid metabolism, further amplifying cardiometabolic risk. Consequently, CVD has emerged as a leading cause of non-cancer mortality among breast cancer survivor.

**Methods:**

We performed a comprehensive narrative review of mechanistic, preclinical, and clinical evidence evaluating the cardiometabolic and oncologic effects of sodium–glucose cotransporter 2 inhibitors (SGLT2i) in the context of breast cancer and cardio-oncology. Literature from experimental models, observational and real-world studies was synthesized to delineate the biological pathways through which SGLT2i may influence cardiovascular risk, metabolic health, and endocrine therapy resistance in HR + BC.

**Main body:**

SGLT2 inhibitors, originally developed as glucose-lowering agents, confer robust cardiovascular and renal protection that extends beyond glycemic control. Emerging evidence indicates that SGLT2i modulate key biological pathways relevant to HR+ BC survivorship, including insulin–IGF-1 signaling, PI3K–Akt–mTOR and AMPK pathways, adipose tissue inflammation, hepatic steatosis, endothelial dysfunction, myocardial energetics, and systemic inflammatory tone. In addition, SGLT2i promote favorable adipose tissue remodeling, with preferential reduction in visceral adiposity and improvement in adipokine profiles, and induce lipid metabolism reprogramming characterized by enhanced fatty acid oxidation and reduced hepatic lipogenesis, contributing to improved cardiometabolic homeostasis. Observational and propensity-matched studies in oncology populations associate SGLT2i use with reduced incidence of heart failure, attenuation of cancer therapy–related cardiac dysfunction, and improved cardiovascular outcomes, including in breast cancer survivors exposed to cardiotoxic therapies. Beyond cardiovascular protection, converging data suggest that SGLT2i may indirectly mitigate or delay resistance to endocrine therapy by suppressing hyperinsulinemia-driven growth signaling, remodeling adipokine and inflammatory networks, and altering tumor–host metabolic interactions.

**Conclusion:**

SGLT2 inhibition represents a promising systems-level strategy at the intersection of cardio-oncology, metabolism, and endocrine resistance in HR+ breast cancer. By simultaneously targeting glucose homeostasis, adipose tissue dysfunction, and lipid metabolism, SGLT2i may improve both cardiovascular outcomes and long-term cancer control. Dedicated prospective trials are urgently needed to define their clinical role in breast cancer survivorship and precision cardio-oncology.

## Introduction

Breast cancer is the most frequently diagnosed malignancy in women worldwide, with hormone receptor–positive (HR+) subtypes accounting for the majority of cases [1]. Endocrine therapies, including aromatase inhibitors (AIs), selective estrogen receptor modulators (SERMs), selective estrogen receptor degraders, and ovarian suppression, remain the cornerstone of treatment across disease stages and are often administered for prolonged durations [2]. While these strategies have substantially improved cancer-specific outcomes, long-term survivorship is increasingly complicated by cardiovascular and metabolic comorbidities. Cardiovascular disease (CVD) has emerged as a leading cause of non-cancer mortality among breast cancer survivors, particularly in women exposed to estrogen-depleting therapies and those with pre-existing or treatment-induced cardiometabolic risk factors [3]. As survival improves, the intersection between endocrine therapy, metabolic dysfunction, and cardiovascular injury has become a critical determinant of long-term outcomes, underscoring the need for integrated survivorship care models that extend beyond tumor control [4, 5] (Table 1). Endocrine therapy contributes to cardiometabolic risk primarily through estrogen deprivation, which disrupts glucose homeostasis and promotes insulin resistance, dyslipidemia, and adverse adipose tissue redistribution. Endocrine therapy contributes to cardiometabolic risk primarily through estrogen deprivation, which disrupts glucose homeostasis and promotes insulin resistance, dyslipidemia, and adverse adipose tissue redistribution. Under physiological conditions, estrogen plays a central role in maintaining metabolic homeostasis by enhancing insulin sensitivity, regulating lipid metabolism, and limiting visceral fat accumulation. Its depletion, whether occurring naturally or induced by endocrine therapy, leads to increased visceral adiposity, reduced peripheral glucose uptake, and compensatory hyperinsulinemia. In addition, estrogen loss is associated with unfavorable lipid changes, including increased low-density lipoprotein cholesterol and reduced high-density lipoprotein levels, as well as activation of pro-inflammatory pathways within adipose tissue. These alterations are closely interconnected: impaired glucose regulation and hyperinsulinemia amplify systemic metabolic stress, leading to endothelial dysfunction, vascular inflammation, and progressive cardiovascular damage. In parallel, changes in lipid metabolism and visceral adiposity further reinforce this metabolic imbalance, creating a pro-atherogenic and pro-inflammatory environment. These changes not only accelerate atherosclerotic and heart failure risk, but also provide metabolic and signaling support for tumor adaptation and potential endocrine resistance [6]. Consequently, breast cancer survivorship represents a paradigmatic setting in which oncologic efficacy, cardiometabolic vulnerability, and treatment resistance converge. Therefore, sodium–glucose cotransporter 2 inhibitors (SGLT2i), originally developed as glucose-lowering agents, have emerged as pleiotropic cardiometabolic modulators with robust cardiovascular and renal benefits that extend beyond glycemic control [7–9]. Growing mechanistic, translational, and real-world clinical evidence suggests that SGLT2 inhibition may address several interconnected pathways relevant to breast cancer survivorship, including insulin–IGF-1 signaling, adipose tissue inflammation, myocardial energetics, and systemic metabolic stress [10].

This state-of-the-art review synthesizes the biological rationale and clinical evidence supporting SGLT2 inhibition in breast cancer patients receiving endocrine therapy, with a focus on cardiometabolic risk modulation and the emerging hypothesis that systemic metabolic reprogramming may influence endocrine resistance. We highlight translational implications, current limitations, and future research priorities at the intersection of cardio-oncology, metabolism, and hormone-dependent breast cancer.

**Table 1:** Cardiometabolic comorbidities in ER+ breast cancer patients receiving endocrine therapy and their clinical consequences

Cardiometabolic Condition	Prevalence / Association in ER+ BC	Pathophysiological Drivers	Clinical Consequences	Implications for Cancer and CV Outcomes
Insulin resistance / Hyperinsulinemia	Highly prevalent; worsens during AI and ovarian suppression therapy	Estrogen deprivation, visceral adiposity, adipose inflammation, reduced insulin sensitivity	Progression to T2DM, endothelial dysfunction, accelerated atherosclerosis	Activation of insulin–IGF-1–PI3K–Akt–mTOR signaling; increased CV risk and potential endocrine resistance
Metabolic syndrome	Up to ~40–50% in long-term survivors	Central obesity, dyslipidemia, hypertension, insulin resistance	Increased risk of ischemic heart disease, HF, and stroke	Higher non-cancer mortality; systemic pro-tumorigenic and pro-inflammatory milieu
Visceral adiposity	Common after menopause or therapy-induced ovarian suppression	Altered fat redistribution, estrogen loss, sedentary behavior	Chronic low-grade inflammation, lipotoxicity, insulin resistance	Leptin-driven ER crosstalk, inflammatory tumor microenvironment, CV remodeling
MASLD (formerly NAFLD)	~25–30% in Western women with obesity/T2DM; enriched in BC survivors	Insulin resistance, hepatic de novo lipogenesis, lipid overload	Progression to NASH, CV events, systemic inflammation	Marker of cardiometabolic risk; contributor to inflammatory and insulin-resistant state
Dyslipidemia	More frequent with aromatase inhibitors than tamoxifen	Loss of estrogen-mediated lipid regulation	Elevated LDL, reduced HDL, vascular dysfunction	Increased ischemic heart disease risk; CV mortality
Hypertension	Increased incidence during endocrine therapy	Vascular stiffness, RAAS activation, metabolic dysfunction	LV hypertrophy, HF risk	Synergistic risk for CTRCD and late HF
Chronic systemic inflammation	Persistent low-grade inflammation common	Adipose cytokine secretion (IL-6, TNF-α), estrogen loss	Endothelial dysfunction, myocardial injury	Promotes CTRCD, fibrosis, and endocrine resistance
Smoking history	Common in older BC populations	Persistent endothelial and oxidative damage	Accelerated vascular disease	Amplifies CV risk during long-term survivorship

## Cardiometabolic vulnerability in HR+ breast cancer on endocrine therapy

Breast cancer survivors receiving endocrine therapy exhibit a distinct and clinically relevant cardiometabolic vulnerability driven by the combined effects of estrogen deprivation, treatment-related lifestyle changes, and pre-existing metabolic risk [11]. Epidemiological studies consistently demonstrate a higher burden of metabolic dysfunction in this population compared with age-matched controls, with implications for both cardiovascular morbidity and long-term cancer outcomes [12] (Table 1). As summarized in Table 1, these cardiometabolic alterations extend beyond isolated metabolic abnormalities and encompass an interconnected network of insulin resistance, visceral adiposity, metabolic dysfunction–associated steatotic liver disease (MASLD), dyslipidemia, hypertension, and chronic inflammatory activation, all of which contribute synergistically to both cardiovascular injury and tumor-promoting signaling pathways.

A highly prevalent condition within this metabolic spectrum is MASLD, a recently redefined clinical entity that replaces and expands the previous concept of nonalcoholic fatty liver disease (NAFLD) [13]. MASLD affects approximately 25–30% of Western women with obesity or type 2 diabetes mellitus (T2DM), and breast cancer survivors frequently fall within this risk spectrum [14]. Estrogen depletion exacerbates hepatic insulin resistance, promotes de novo lipogenesis, and accelerates intrahepatic fat accumulation, contributing to systemic inflammation and heightened cardiovascular risk [15]. Endocrine therapy is also associated with central adiposity and preferential visceral fat accumulation, driven by menopause or therapy-induced ovarian suppression. Visceral adipose tissue is metabolically active and characterized by increased secretion of pro-inflammatory cytokines, including tumor necrosis factor-α and interleukin-6, which promote insulin resistance, endothelial dysfunction, and chronic low-grade inflammation [16]. This visceral adipose expansion is closely linked to alterations in glucose homeostasis. Increased lipolytic activity within visceral fat depots leads to enhanced release of free fatty acids into the portal circulation, promoting hepatic insulin resistance and increased gluconeogenesis. In parallel, adipose tissue inflammation further impairs insulin signaling through serine phosphorylation of insulin receptor substrate proteins and disruption of the PI3K–Akt pathway. These mechanisms reduce peripheral glucose uptake in skeletal muscle and drive compensatory hyperinsulinemia. Over time, this metabolic state progresses toward impaired fasting glucose and overt hyperglycemia, even in individuals without pre-existing diabetes. Clinically, these alterations contribute to elevated HbA1c levels and reinforce the link between endocrine therapy–induced metabolic dysfunction and increased cardiovascular risk. These alterations are compounded by the loss of estrogen’s vasoprotective and anti-inflammatory effects. As a result, up to 40–50% of breast cancer survivors meet criteria for metabolic syndrome, encompassing hypertension, dyslipidemia, impaired fasting glucose, and central obesity [17]. Insulin resistance is particularly pronounced and is often reflected by elevated HOMA-IR indices and modest but clinically meaningful increases in glycated hemoglobin (HbA1c), even below diabetic thresholds [18, 19]. Chronic hyperinsulinemia and activation of the insulin–IGF-1 axis amplify atherogenic processes and mitogenic signaling, linking cardiometabolic disease with both cardiovascular events and tumor progression (Table 1). Importantly, Table 1 also highlights how these metabolic abnormalities frequently coexist and reinforce one another during long-term survivorship, thereby generating a persistent pro-inflammatory and pro-atherogenic milieu that may simultaneously favor CTRCD susceptibility and endocrine-resistant tumor adaptation.

Recognizing this vulnerability, the 2021 European Society of Cardiology (ESC) Guidelines on cardiovascular disease prevention classify breast cancer survivors receiving endocrine therapy as a population at increased long-term cardiovascular risk [20, 21]. The guidelines emphasize structured baseline cardiovascular assessment, early risk factor modification, and longitudinal surveillance, particularly in patients with metabolic comorbidities or prolonged exposure to aromatase inhibitors [22]. These recommendations align with contemporary cardio-oncology principles advocating for integrated, preventive strategies across the cancer care continuum [23]. Collectively, hormone receptor–positive breast cancer survivors undergoing endocrine therapy represent a high-priority group for targeted cardiometabolic intervention, providing a strong clinical foundation for evaluating therapies capable of simultaneously addressing metabolic dysfunction, cardiovascular risk, and treatment-related vulnerability (Table 1).

## Biological rationale and hypothesis-generating mechanisms for SGLT2 inhibition in HR+ breast cancer survivorship

SGLT2 inhibitors (SGLT2i), initially developed as glucose-lowering agents, have emerged as pleiotropic cardiometabolic modulators with established cardiovascular and renal protective effects extending beyond glycemic control [24, 25]. Because breast cancer survivors receiving endocrine therapy frequently exhibit insulin resistance, visceral adiposity, MASLD, hypertension, and elevated cardiovascular risk, SGLT2i represent a biologically attractive therapeutic strategy in this setting [26, 27]. Rather than acting through a single pathway, SGLT2i influence interconnected metabolic, inflammatory, hemodynamic, and bioenergetic mechanisms that are increasingly recognized as contributors to both cardiovascular remodeling and systemic metabolic dysfunction. Many of these mechanisms have been extensively reviewed elsewhere, including in dedicated cardio-oncology reviews [10]. Therefore, the present section focuses primarily on mechanisms that may be particularly relevant to endocrine therapy–associated cardiometabolic vulnerability and to the emerging, but still unproven, hypothesis that systemic metabolic modulation could influence endocrine resistance in HR+ breast cancer.

### Metabolic reprogramming, insulin signaling, and adipose tissue remodeling

SGLT2i induce insulin-independent glucosuria through inhibition of glucose reabsorption in the proximal renal tubule, leading to reductions in fasting glucose, HbA1c, circulating insulin levels, and HOMA-IR indices [28–32]. These effects are particularly relevant in postmenopausal breast cancer survivors receiving estrogen-depleting therapies, a population characterized by high rates of insulin resistance and hyperinsulinemia. Given the established role of insulin and IGF-1 signaling in activating the PI3K–Akt–mTOR pathway, SGLT2i-mediated attenuation of systemic hyperinsulinemia has generated interest as a potential upstream metabolic strategy capable of indirectly modulating pathways implicated in endocrine resistance and cardiovascular remodeling [33–37]. However, it is important to emphasize that current evidence remains indirect. To date, no preclinical or clinical study has directly demonstrated that SGLT2i improve sensitivity to aromatase inhibitors, SERMs, or SERDs in HR+ breast cancer models or patients. SGLT2i exert important effects on adipose tissue biology and systemic metabolic homeostasis, mechanisms that may be particularly relevant in breast cancer survivors receiving endocrine therapy, a population characterized by visceral adiposity, insulin resistance, and chronic low-grade inflammation [38]. Clinical imaging and metabolic studies demonstrate that SGLT2 inhibitor therapy induces preferential reductions in visceral and hepatic fat depots relative to total body weight loss, suggesting a qualitative remodeling of adipose tissue distribution rather than a purely quantitative reduction in adiposity [39]. This metabolic remodeling is accompanied by favorable changes in adipokine signaling, including increased adiponectin levels and reductions in leptin and resistin concentrations [40]. These shifts may contribute to restoration of a more insulin-sensitive and anti-inflammatory metabolic environment, with attenuation of macrophage infiltration, NLRP3 inflammasome activation, and adipose tissue–derived cytokine signaling [41]. Such mechanisms are particularly relevant in HR+ breast cancer survivors exposed to estrogen deprivation, where visceral adiposity and adipose inflammation contribute not only to cardiometabolic dysfunction but also to systemic insulin–IGF-1 activation and endocrine-independent growth signaling. In hepatic tissue, SGLT2 inhibition promotes β-oxidation of fatty acids, suppresses de novo lipogenesis, and reduces intrahepatic triglyceride accumulation through mechanisms involving AMPK activation, suppression of sterol regulatory element-binding protein-1c (SREBP-1c), and reduced intracellular glucose flux [42, 43]. These effects translate into improvements in hepatic steatosis and MASLD severity, conditions increasingly recognized as contributors to systemic inflammation, endothelial dysfunction, and long-term cardiovascular risk in breast cancer survivorship. Although these metabolic and adipose tissue effects provide a biologically plausible framework linking SGLT2 inhibition to improved cardiometabolic resilience in endocrine therapy–treated patients, direct evidence demonstrating that adipose remodeling induced by SGLT2i alters endocrine responsiveness or delays endocrine resistance in HR+ breast cancer remains unavailable.

### Cardiorenal hemodynamics and myocardial energetics

**Fig. 1:**
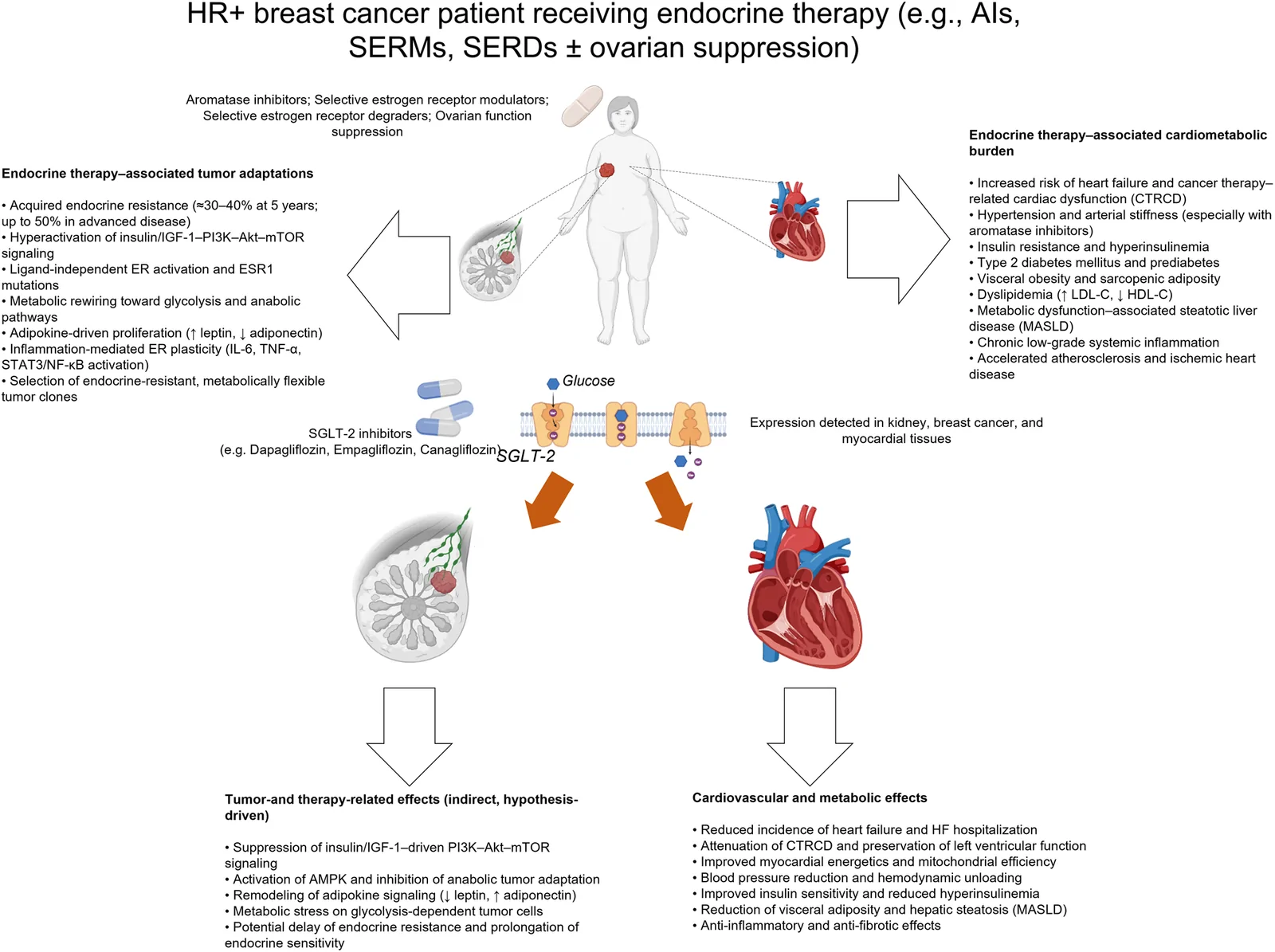
Endocrine therapy–associated cardiometabolic vulnerability and potential systemic benefits of SGLT2 inhibition in hormone receptor–positive breast cancer. Endocrine therapies for hormone receptor–positive breast cancer promote insulin resistance, visceral adiposity, and adverse cardiovascular remodeling, increasing the risk of metabolic dysfunction, cancer therapy–related cardiac dysfunction, and heart failure. Through renal glucose handling and systemic metabolic reprogramming, SGLT2i improve glycemic and insulin homeostasis, attenuate cardiometabolic stress, and may indirectly modulate tumor–host metabolic interactions, supporting cardiovascular protection and long-term survivorship care

The cardiovascular benefits of SGLT2i are among the most robust and reproducible findings associated with this drug class [44]. Through combined osmotic diuresis and natriuresis, SGLT2i reduce intravascular volume, ventricular preload, and systemic afterload, resulting in sustained reductions in systolic and diastolic blood pressure without reflex tachycardia or neurohormonal activation [45]. These hemodynamic effects are particularly relevant in breast cancer survivors receiving aromatase inhibitors, in whom estrogen deprivation is associated with increased arterial stiffness, endothelial dysfunction, and hypertension [46]. Additional mechanistic insights have emerged regarding the interaction between SGLT2 inhibition and sodium–hydrogen exchanger (NHE) isoforms, which may contribute to both renal and cardiac effects. At the renal level, SGLT2 is functionally and anatomically co-localized with NHE3 in the proximal tubule, and pharmacological SGLT2 inhibition has been shown to attenuate NHE3-mediated sodium reabsorption, thereby enhancing natriuresis independently of glucosuria [47, 48]. This interaction may partially explain the consistent hemodynamic benefits observed with SGLT2i, including reductions in plasma volume and blood pressure. At the cardiac level, where SGLT2 expression is negligible, SGLT2 inhibitors have been proposed to exert off-target effects through inhibition of the sodium–hydrogen exchanger isoform 1 (NHE1) [49]. NHE1 inhibition leads to a reduction in intracellular Na^+^ concentration, which in turn limits Ca^2 +^ overload via reverse-mode Na^+^ /Ca^2 +^ exchange, thereby mitigating key drivers of cardiomyocyte injury, including mitochondrial dysfunction, oxidative stress, and activation of Ca^2 +^ -dependent signaling pathways. Notably, intracellular Na^+^ and Ca^2 +^ dysregulation and CaMKII activation represent central mechanisms in anthracycline-induced cardiotoxicity, suggesting a biologically plausible overlap between NHE1 modulation and pathways implicated in cancer therapy–related cardiac dysfunction (CTRCD), although this specific link has not yet been directly demonstrated in dedicated experimental models. Recent experimental evidence further supports the relevance of this pathway, demonstrating that empagliflozin attenuates cardiac hypertrophy, fibrosis, and dysfunction in SGLT2 knockout models via mechanisms involving the NHE1–nitric oxide axis, indicating that these cardioprotective effects may occur independently of canonical SGLT2 inhibition [50]. However, it is important to acknowledge that the extent to which SGLT2 inhibitors directly inhibit NHE1 remains an area of ongoing debate. While several studies support a direct inhibitory effect, others suggest that the observed changes in intracellular sodium handling may be indirect or context-dependent [51, 52]. Accordingly, the NHE1/NHE3 axis should be considered a plausible but not yet fully resolved component of the broader mechanistic framework underlying SGLT2i-mediated cardioprotection. Beyond hemodynamic unloading, SGLT2 inhibition induces profound changes in myocardial energy metabolism. By promoting a mild, chronic shift toward ketone body utilization and activating energy-sensing pathways, including the SIRT1–PGC-1α–FGF21 axis, SGLT2i enhance mitochondrial efficiency, reduce reactive oxygen species generation, and improve myocardial ATP production per unit of oxygen consumed (Fig. 1) [53]. This bioenergetic reprogramming confers resistance to metabolic stress, limits maladaptive hypertrophy, and attenuates fibrotic remodeling. Such mechanisms are of particular relevance in patients exposed to anthracyclines or HER2-targeted therapies, where mitochondrial dysfunction and oxidative injury are central drivers of cancer therapy–related cardiac dysfunction.

### Inflammatory remodeling, cardioprotection, and emerging tumor-modulating effects

Chronic inflammation and oxidative stress represent shared pathogenic substrates linking cardiometabolic dysfunction, cancer progression, and therapy-induced cardiovascular injury [54, 55]. SGLT2i consistently reduce circulating levels of pro-inflammatory mediators, including interleukin-6 (IL-6), tumor necrosis factor-α (TNF-α), and C-reactive protein (CRP), effects that appear partly independent of glucose lowering and closely associated with improvements in adipose tissue inflammation, endothelial function, and mitochondrial homeostasis [56, 57]. Concomitantly, SGLT2 inhibition attenuates systemic and myocardial oxidative stress, as reflected by reductions in lipid peroxidation markers such as malondialdehyde and 8-isoprostane [58]. At the cellular level, these effects are associated with reduced activation of pro-fibrotic signaling pathways, including TGF-β, attenuation of cardiomyocyte apoptosis, and preservation of myocardial structure and function in experimental models of anthracycline-induced cardiotoxicity [59].

Beyond cardioprotection, accumulating preclinical evidence suggests that SGLT2i may exert indirect tumor-modulating effects through systemic metabolic and inflammatory reprogramming [60]. In breast cancer cell lines, SGLT2 inhibition has been associated with reduced glucose uptake, induction of energetic stress, cell-cycle arrest, and apoptosis, partly mediated through suppression of Akt/mTOR signaling and activation of AMPK-dependent pathways [61]. In parallel, systemic reductions in insulin and IGF-1 concentrations may deprive tumor cells of key proliferative stimuli, while increased ketone body availability may generate a metabolically unfavorable environment for highly glycolytic cancer phenotypes [62]. SGLT2i-mediated reductions in leptin signaling and adipose tissue inflammation may additionally influence the tumor microenvironment, particularly in obesity-associated and estrogen-deprived conditions frequently observed in HR+ breast cancer survivors. However, it is important to emphasize that the currently available evidence remains largely preclinical and indirect. Table 2 further illustrates that most available oncology-related evidence derives from metabolic or antiproliferative experimental models rather than studies specifically assessing endocrine therapy responsiveness or resistance-related endpoints. Existing studies primarily demonstrate generic antiproliferative or metabolic effects of SGLT2i in breast cancer models and do not directly evaluate modulation of responsiveness to endocrine therapies such as aromatase inhibitors, SERMs, or SERDs [68, 69, 78]. Therefore, the hypothesis that SGLT2 inhibition may delay endocrine resistance should currently be interpreted as a biologically plausible and testable conceptual framework rather than an established therapeutic paradigm. Future translational studies should specifically investigate whether SGLT2i influence endocrine responsiveness in clinically relevant HR+ breast cancer models, including aromatase inhibitor–resistant and ESR1-mutant cell lines, patient-derived organoids, and adipocyte–tumor co-culture systems capable of reproducing the metabolic and inflammatory microenvironment associated with endocrine therapy. Integration of longitudinal metabolic phenotyping, insulin signaling analysis, and resistance-specific endpoints may help determine whether systemic metabolic reprogramming induced by SGLT2 inhibition can meaningfully alter the biology of endocrine resistance.

Collectively, SGLT2i represent a potentially attractive therapeutic strategy in breast cancer survivors characterized by cardiometabolic vulnerability, systemic inflammation, and prior exposure to cardiotoxic therapies. These studies have been conducted across heterogeneous clinical contexts, including patients receiving anthracycline-based chemotherapy, HER2-targeted therapies such as trastuzumab, and other cardiotoxic regimens, as well as broader oncology populations with coexisting cardiometabolic disease. To facilitate interpretation of the drug-specific evidence discussed, Table 2 summarizes the principal SGLT2 inhibitors evaluated in preclinical breast cancer models, cardio-oncology cohorts, and cardiovascular outcome trials relevant to breast cancer survivorship. As detailed in Table 2, the available evidence spans multiple translational levels, ranging from experimental tumor and cardiotoxicity models to observational cardio-oncology cohorts and ongoing prospective clinical trials. Importantly, the table highlights the predominance of indirect and nonrandomized evidence, while also illustrating the growing convergence between mechanistic cardioprotection, systemic metabolic modulation, and emerging oncology-specific applications of SGLT2 inhibition. Across these datasets, SGLT2i exposure is consistently associated with lower rates of incident HF, HF hospitalization, and composite cardiovascular events, with emerging signals suggesting a potential role in primary prevention of CTRCD, particularly in populations characterized by cardiometabolic vulnerability (type 2 diabetes mellitus, insulin resistance, metabolic syndrome, chronic kidney disease, MASLD) and/or exposure to anthracyclines and HER2-targeted therapy [79–82]. Notably, Table 2 also underscores the heterogeneity of the currently available literature, which includes differences in tumor populations, cardiotoxic exposures, comparator groups, and endpoint definitions. This variability likely contributes to the discordant magnitude of observed benefit across studies and reinforces the need for dedicated randomized cardio-oncology trials specifically designed for breast cancer survivorship settings Importantly, the current evidence base remains dominated by nonrandomized designs; therefore, although the observed associations are biologically coherent and clinically encouraging, causal inference remains limited by residual confounding, treatment-selection bias, and survivorship or immortal-time bias.

**Table 2:** Drug-specific evidence for SGLT2 inhibitors. This table summarizes the principal SGLT2 inhibitors evaluated in preclinical breast cancer models, cardio-oncology studies, and cardiovascular outcome trials relevant to breast cancer survivorship. Evidence is strongest for heart failure and cardiorenal protection in non-oncology populations, whereas breast cancer–specific and endocrine therapy–specific data remain limited. Preclinical tumor-modulating effects should be interpreted as hypothesis-generating and do not establish direct anticancer or endocrine-sensitizing efficacy

SGLT2 inhibitor	Evidence type	Population / model	Oncology or cardiovascular models	Main preclinical and clinical findings	References
Dapagliflozin	Preclinical / translational	Breast cancer cell lines and experimental tumor models	Tumor metabolic stress and growth signaling	Reduced glucose uptake, induced metabolic stress, activated AMPK, inhibited mTOR signaling, and promoted cell-cycle arrest/apoptosis in breast cancer models.	[61, 62]
Dapagliflozin	Preclinical cardio-oncology	Experimental models of anthracycline and HER2-targeted therapy–related cardiotoxicity	Cancer therapy–related cardiac injury	Attenuated oxidative stress, inflammation, and myocardial injury in models relevant to anthracycline/HER2 therapy–associated cardiotoxicity.	[58]
Dapagliflozin	Randomized trial design / ongoing clinical trial	Early-stage breast cancer patients receiving neoadjuvant anthracycline-based chemotherapy ± trastuzumab	Primary prevention of chemotherapy-induced cardiotoxicity	PROTECT is designed to evaluate whether dapagliflozin reduces anthracycline- and/or trastuzumab-associated cardiotoxicity in breast cancer patients.	[63]
Dapagliflozin	Cardiovascular outcome trial	Patients with heart failure with reduced ejection fraction	Heart failure prevention and treatment framework	Reduced worsening heart failure and cardiovascular death, supporting cardioprotection independent of diabetes status and informing cardio-oncology extrapolation.	[64]
Empagliflozin	Preclinical cardio-oncology	Non-diabetic murine model of doxorubicin-induced cardiotoxicity	Anthracycline-induced myocardial injury	Improved myocardial strain, reduced cardiac fibrosis and inflammatory cytokines, and preserved cardiac structure/function after doxorubicin exposure.	[59]
Empagliflozin	Prospective clinical study	Breast cancer patients scheduled for anthracycline-based chemotherapy	Primary prevention of anthracycline-induced cardiotoxicity	EMPACARD-PILOT reported a favorable association between empagliflozin use and preservation of cardiac function / reduced CTRCD signal in high-risk breast cancer patients.	[65]
Empagliflozin	Cardiovascular outcome trials	Patients with HFrEF and HFpEF	Heart failure outcomes	Reduced heart failure hospitalization and improved cardiorenal outcomes across ejection-fraction phenotypes, supporting broad HF protection.	[66, 67]
Canagliflozin	Preclinical oncology	Breast cancer cell lines / xenograft models	Tumor glucose uptake and AMPK–mTOR signaling	Reduced breast cancer cell proliferation and colony formation, activated AMPK, suppressed mTOR/p70S6K signaling, and induced cell-cycle arrest/apoptosis.	[61]
Canagliflozin	Preclinical oncology / chemotherapy interaction	MCF-7 breast cancer cells exposed to doxorubicin	Chemosensitivity and tumor cell viability	Evaluated in combination with doxorubicin; reported modulation of breast cancer cell viability and potential chemosensitizing effects in vitro.	[68]
Ipragliflozin	Preclinical oncology	MCF-7 breast cancer model	Breast cancer cell proliferation	Attenuated breast cancer cell proliferation in experimental models, supporting drug-class exploration beyond dapagliflozin and empagliflozin.	[69]
SGLT2i class effect	Propensity-matched observational study	Patients with diabetes receiving cancer therapy	Primary prevention of CTRCD	SGLT2i use was associated with lower 12-month CTRCD risk and fewer HF exacerbations, supporting potential cardioprotection in oncology populations.	[26]
SGLT2i class effect	Retrospective clinical study	Older diabetic women with early-stage breast cancer treated with anthracycline and/or trastuzumab	Breast cancer–specific cardioprotection	No significant cardiovascular benefit over other oral antidiabetic comparators, providing an important counterbalancing clinical signal.	[70]
SGLT2i class effect	Systematic reviews / meta-analyses	Cancer patients and survivors; cancer patients with diabetes; anthracycline-treated patients	HF, CTRCD, mortality, and CV outcomes	Overall signals suggest lower HF incidence/HF hospitalization and improved cardiovascular outcomes, but evidence remains largely observational and heterogeneous.	[71–77]

## Clinical evidence of SGLT2i in oncology and breast cancer

A recent systematic review and meta-analysis focusing on HF outcomes in cancer patients and survivors reported that SGLT2i use is associated with a significant reduction in incident/new HF, with an effect estimate in pooled analysis consistent with a clinically meaningful risk reduction (reported RR ~0.29 in the analysis of studies from 2022 to 2024) [71, 72].

While the underlying studies span multiple tumor types and chemotherapy exposures, subgroup signals suggest that benefit may be particularly prominent in cohorts enriched for anthracycline exposure and baseline cardiometabolic disease [73].

A separate systematic review/meta-analysis centered on cancer patients with T2DM undergoing chemotherapy evaluated SGLT2i versus non-SGLT2i comparators and incorporated subgroup analyses (including patients without baseline HF and those treated with anthracyclines). This work supports an association between SGLT2i exposure and improved cardiovascular outcomes in these high-risk contexts, reinforcing the plausibility of a class effect extending into oncology care pathways [74–76].

A 2025 systematic review/meta-analysis specifically examining anthracycline-treated cancer patients reported lower all-cause mortality in those receiving SGLT2i compared with controls, further supporting the hypothesis that SGLT2i may mitigate downstream clinical deterioration in regimens with well-established cardiotoxic potential [77].

Across meta-analytic syntheses, the most reproducible clinical signal pertains to HF-related endpoints, which align with the strongest established benefits of SGLT2i in non-oncology cardiology populations. However, literature is still limited by endpoint heterogeneity (ICD-coded HF vs adjudicated HF; variable definitions of CTRCD), inconsistent reporting of cumulative anthracycline dose/HER2 exposure, and incomplete adjustment for cardio-protective co-therapies (ACEi/ARB/ARNI, beta-blockers, MRA, statins) and baseline echocardiographic phenotype (Fig. 1).

### Prevention of cancer therapy–related cardiac dysfunction

One of the most influential contemporary datasets addressing primary prevention in oncology populations exposed to cardiotoxic therapies, particularly anthracycline-based regimens and HER2-targeted treatments, is a large propensity-matched analysis evaluating SGLT2i prescription and subsequent CTRCD risk at 12 months [26]. In this study, SGLT2i exposure was associated with a lower risk of CTRCD (HR ~ 0.76, 95% CI 0.69–0.84), alongside reductions in HF exacerbations and all-cause mortality, suggesting that SGLT2i may impact both intermediate CTRCD and downstream clinical endpoints in cancer populations (Fig. 1).

Professional society commentary has highlighted these findings as a substantive step forward while also emphasizing the need for prospective randomized confirmation.

Importantly, recent prospective and real-world data have begun to more directly address the role of SGLT2 inhibitors in cardiotoxicity prevention within breast cancer populations. The EMPACARD-PILOT study [65] to our knowledge the first prospective investigation specifically evaluating SGLT2 inhibition for the prevention of anthracycline-induced cardiotoxicity in breast cancer patients, provides an initial signal supporting a potential cardioprotective effect in this high-risk setting. Although limited by sample size and exploratory design, this study represents a critical step toward moving beyond associative evidence and establishing a prospective clinical framework for SGLT2i use in cardio-oncology. The clinical relevance and interpretative boundaries of these findings have been further contextualized by accompanying editorial and expert commentary [83, 84], which emphasize both the mechanistic plausibility and the current limitations of the evidence base, including the need for standardized CTRCD definitions, longer follow-up, and randomized validation. These perspectives underscore that, while early signals are encouraging, definitive conclusions regarding causality and clinical implementation remain premature. Conversely, emerging real-world evidence highlights the heterogeneity of observed effects. A recent retrospective study by Liu et al. [70], conducted in older diabetic women with early-stage breast cancer treated with anthracyclines and/or trastuzumab, did not demonstrate a significant reduction in cardiovascular events with SGLT2 inhibitor use compared with other oral antidiabetic therapies. This neutral finding is particularly informative, as it suggests that cardioprotective effects may be context-dependent and influenced by patient selection, competing risk factors, comparator therapies, and methodological considerations inherent to observational designs. Taken together, these data reinforce the concept that, although SGLT2 inhibitors represent a biologically compelling and clinically promising strategy for CTRCD prevention, the current evidence remains heterogeneous and largely non-definitive. The integration of early prospective signals with discordant real-world findings further highlights the urgent need for adequately powered, randomized controlled trials specifically designed in breast cancer populations. Ongoing studies, including the PROTECT trial [63], evaluating dapagliflozin for the prevention of anthracycline- and trastuzumab-associated cardiotoxicity in early-stage breast cancer, are expected to provide critical insights to define the magnitude and consistency of cardioprotective effects in this setting.

CTRCD is increasingly conceptualized as a spectrum encompassing early myocardial injury (biomarker rise), impaired myocardial energetics/mitochondrial dysfunction, microvascular dysfunction, inflammation, and subsequent remodeling [85–87]. The observed real-world association between SGLT2i use and lower CTRCD risk is mechanistically coherent with established SGLT2i biology (hemodynamic unloading, improved cardiac energetics, anti-inflammatory/anti-fibrotic effects), but the extent to which these mechanisms translate into clinically meaningful prevention across distinct cardiotoxic agents (anthracyclines vs HER2-targeted therapy vs immune checkpoint inhibitors) is not yet resolved (Fig. 1) [88].

### Evidence in breast cancer and endocrine therapy settings

Although no completed large RCT has yet definitively established SGLT2i for CV prevention specifically in breast cancer patients on endocrine therapy, the evidence is strengthening in adjacent, clinically relevant populations. Available evidence in breast cancer is largely derived from patients treated with anthracyclines and/or HER2-targeted therapies, which remain the principal drivers of cancer therapy–related cardiac dysfunction in this population.

In systematic reviews/meta-analyses of cancer cohorts, the magnitude of HF benefit has been reported as particularly pronounced in breast cancer patients receiving anthracycline-based chemotherapy, suggesting potential regimen-specific or population-specific enrichment effects [89].

This is clinically relevant because anthracycline exposure and HER2-targeted therapy remain common drivers of late HF risk in long-term breast cancer survivorship, and their cardiotoxicity may intersect with endocrine therapy–related metabolic deterioration.

Observational propensity score–matched cohort studies in patients with cancer and type 2 diabetes, including breast cancer populations, have associated SGLT2i exposure with lower rates of heart failure hospitalization and improved overall survival compared with non-SGLT2i therapies, although these analyses remain vulnerable to treatment-selection bias and residual confounding [90]. Nonetheless, the overall directionality of effect is consistent with the established cardiovascular benefits observed in large non-oncology cardiovascular outcome trials, supporting biological plausibility and external validity. Figure 1 conceptually integrates these observations by linking endocrine therapy–associated metabolic dysfunction with the principal systemic pathways potentially targeted by SGLT2 inhibition, including insulin–IGF-1 signaling, adipose inflammation, myocardial energetics, and CTRCD-related remodeling.

The breast cancer endocrine therapy setting is characterized by (i) adverse lipid shifts, (ii) increased visceral adiposity and insulin resistance, (iii) higher incidence of hypertension and vascular stiffness, particularly with aromatase inhibitors and ovarian suppression, thereby creating a “substrate” where SGLT2i biology would be expected to yield outsized benefit [91]. As illustrated in Fig. 1, endocrine therapy creates a multidimensional cardiometabolic and inflammatory milieu characterized by insulin resistance, visceral adiposity, dyslipidemia, endothelial dysfunction, and tumor metabolic adaptation, thereby providing a biologically coherent framework through which SGLT2 inhibition could simultaneously influence cardiovascular vulnerability and systemic metabolic stress. To date, however, the endocrine therapy population remains underrepresented in cardio-oncology outcome datasets, and trials explicitly designed around this question are still needed. Large HF outcome trials (e.g., DAPA-HF, EMPEROR-Reduced, EMPEROR-Preserved) [64, 66, 67] generally excluded active malignancy and recent cancer diagnoses; nonetheless, post-hoc analyses in participants with prior cancer histories have generally shown preserved relative risk reduction for HF hospitalization and CV mortality comparable to the overall trial populations, without clear safety signals for cancer recurrence. These analyses help support biological plausibility and general safety but do not substitute for dedicated cardio-oncology RCTs, because cancer treatment exposure, stage, and active therapy-related inflammatory states are not adequately captured [92] (Fig. 1). Importantly, Fig. 1 also highlights the hypothesis-generating nature of the proposed framework, emphasizing that many of the potential tumor-modulating effects of SGLT2 inhibition remain indirect, systemic, and incompletely validated in endocrine-resistant HR+ breast cancer models.

## Metabolic reprogramming and endocrine resistance: a unifying framework

**Fig. 2:**
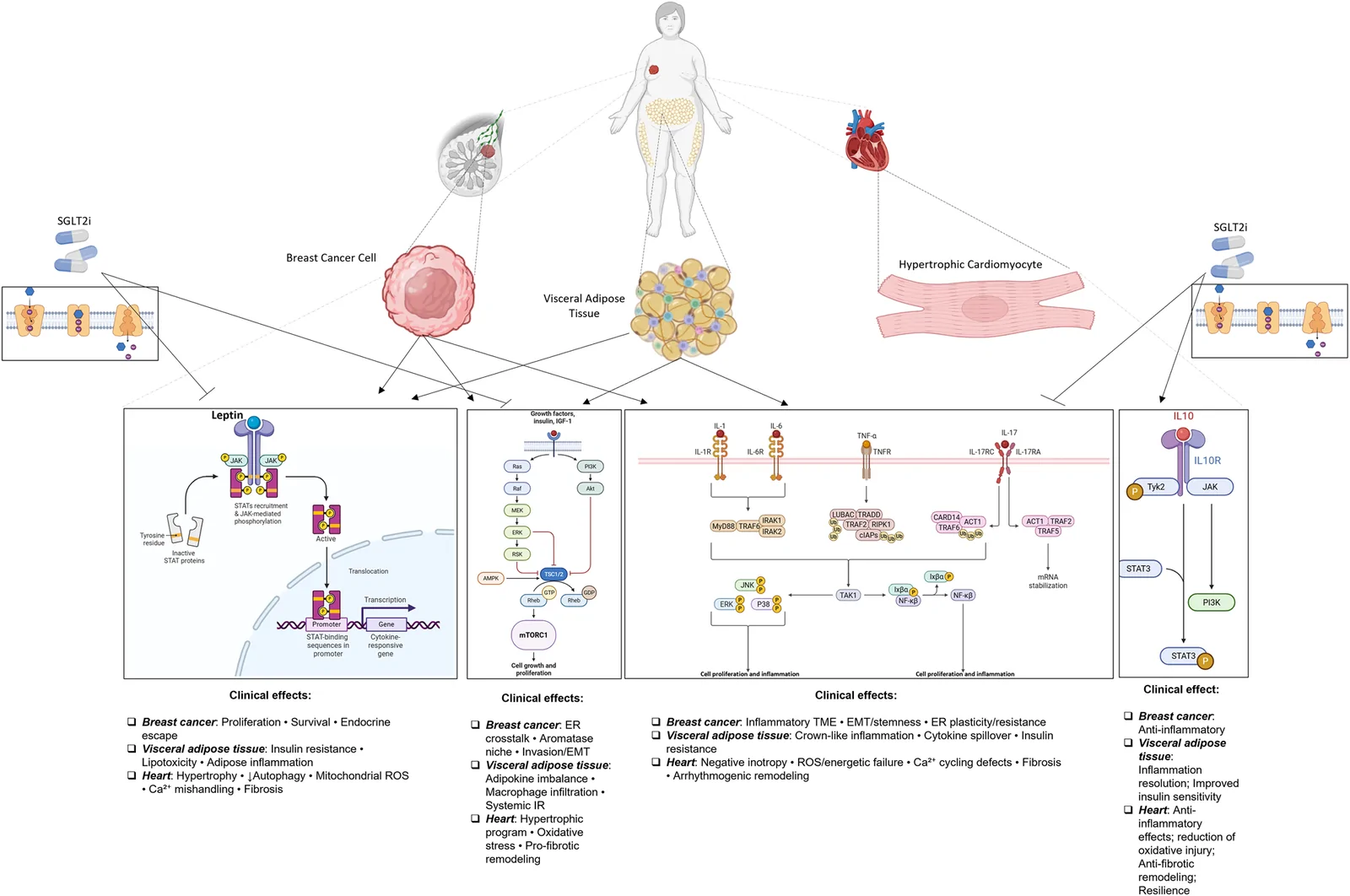
Integrated metabolic and inflammatory signaling linking endocrine therapy, cardiometabolic dysfunction, and tumor adaptation in hormone receptor–positive breast cancer. Endocrine therapy induces systemic insulin resistance, visceral adipose tissue dysfunction, and chronic inflammation, which converge on breast cancer cells and cardiomyocytes. Insulin/IGF-1, leptin, and pro-inflammatory cytokines activate PI3K–Akt–mTOR, JAK/STAT3, NF-κB, and MAPK pathways, promoting endocrine resistance, metabolic rewiring, myocardial hypertrophy, energetic dysfunction, and adverse cardiac remodeling. Activation of the IL-10 axis counterbalances inflammatory signaling and supports metabolic and myocardial resilience. Through renal glucose handling and systemic metabolic reprogramming, SGLT2i may attenuate these maladaptive pathways, providing cardiometabolic protection and potentially delaying endocrine resistance. The figure represents a hypothesis-generating translational framework integrating currently available mechanistic and preclinical evidence rather than a clinically validated therapeutic model

One of the defining features of endocrine-resistant breast cancer is metabolic plasticity [93]. Long-term estrogen deprivation induces a shift from ER-dependent transcriptional programs toward alternative survival pathways that rely heavily on insulin/IGF-1 signaling, PI3K–Akt–mTOR activation, and enhanced glycolytic flux [94, 95] (Table 3). As summarized in Table 3, these pathways are not restricted to tumor biology alone but simultaneously intersect with mechanisms involved in myocardial remodeling, mitochondrial dysfunction, inflammation, fibrosis, and CTRCD susceptibility, thereby reinforcing the concept of a shared cardiometabolic signaling network linking endocrine resistance and cardiovascular vulnerability. Hyperinsulinemia and systemic insulin resistance, frequent consequences of menopause, ovarian suppression, and AI therapy, further amplify these pathways, creating a permissive metabolic environment for endocrine escape [96]. SGLT2i reduce systemic glucose availability and circulating insulin levels through insulin-independent glucosuria, leading to sustained attenuation of insulin and IGF-1 signaling [97] (Table 2). Preclinical models consistently demonstrate that suppression of insulin/IGF-1 reduces phosphorylation of Akt and downstream mTOR activity, pathways strongly implicated in resistance to AIs and SERMs [98]. Table 3 further illustrates how several of these signaling cascades, including PI3K–Akt–mTOR, AMPK, JAK/STAT3, NF-κB, and oxidative stress pathways, may represent convergent biological nodes through which systemic metabolic interventions could theoretically influence both endocrine-resistant tumor adaptation and cardiovascular remodeling. Given that PI3K–Akt–mTOR hyperactivation is one of the most common molecular drivers of endocrine resistance, SGLT2i-mediated metabolic modulation may function as an upstream, systemic strategy to blunt this adaptive signaling, thereby potentially contributing to preservation of endocrine sensitivity (Fig. 2) [99]. However, as reflected in Table 3, the majority of these proposed interactions are currently inferred from pathway overlap and systemic metabolic biology rather than from direct experimental demonstration of endocrine sensitization induced by SGLT2 inhibition in HR+ breast cancer models.

**Table 3:** Molecular pathways linking endocrine resistance and cancer therapy–related cardiac dysfunction (CTRCD), and modulatory effects of SGLT2 inhibitors

Pathway	Primary Activation Source	Effect in Tumor Cells (ER+ BC)	Effect in Cardiovascular system	Effect of SGLT2 Inhibition
Insulin / IGF-1 → PI3K–Akt–mTORC1	Hyperinsulinemia, metabolic syndrome	ER-independent proliferation; endocrine escape; anabolic rewiring	Pathological hypertrophy; suppressed autophagy; mitochondrial dysfunction; fibrosis	↓ Insulin/IGF-1 signaling; ↓ mTORC1 activation; restoration of metabolic balance
AMPK signaling	Energy stress, reduced glucose availability	Inhibition of anabolic growth; increased endocrine sensitivity	Improved mitochondrial efficiency; enhanced autophagy; cardioprotection	↑ AMPK activation; favors catabolic, stress-resilient phenotypes
Leptin → JAK/STAT3	Visceral adiposity, adipose inflammation	Aromatase induction; ER crosstalk; migration and invasion	Pro-hypertrophic and pro-fibrotic signaling; oxidative stress	↓ Leptin levels; adipose remodeling; attenuation of leptin-driven signaling
IL-6 / STAT3	Chronic inflammation, adipose cytokine spillover	EMT, stemness, ER plasticity, therapy resistance	Inflammatory remodeling; contractile dysfunction	↓ Systemic inflammation; indirect suppression of STAT3 overactivation
TNF-α / IL-1β → NF-κB	Adipose tissue macrophages; therapy-induced inflammation	Pro-survival inflammatory niche; resistance signaling	Negative inotropy; fibrosis; arrhythmogenic remodeling	↓ Cytokine burden; reduced NF-κB–driven injury
IL-17 axis	Chronic immune activation	Fibrosis-prone, inflammatory tumor microenvironment	Fibrotic myocardial remodeling	Indirect attenuation via inflammatory resolution
Oxidative stress / mitochondrial dysfunction	Metabolic overload; cardiotoxic therapies	Adaptive survival under stress	ROS accumulation; energetic failure; CTRCD	↓ ROS; improved mitochondrial energetics
IL-10 → STAT3 (anti-inflammatory)	Resolution-phase immune signaling	Anti-inflammatory effects	Cytoprotection; reduced fibrosis; inflammation resolution	↑ IL-10 signaling; promotes myocardial and metabolic resilience

### PI3K–Akt–mTOR axis and crosstalk with estrogen receptor signaling

Endocrine resistance frequently arises from ligand-independent ER activation or ER bypass via growth factor receptor signaling [100]. Activation of PI3K–Akt–mTOR not only drives proliferation independently of estrogen but also phosphorylates ERα at activating sites, rendering tumor cells less dependent on estrogenic stimulation (Fig. 2) [101]. Figure 2 illustrates how this signaling network extends beyond tumor cells and intersects with adipose tissue inflammation, systemic insulin resistance, and myocardial remodeling, supporting the concept of a shared cardiometabolic substrate underlying both endocrine resistance and cardiovascular vulnerability. This crosstalk explains why PI3K and mTOR inhibitors have demonstrated clinical benefit in endocrine-resistant disease. SGLT2i indirectly target this axis by reducing upstream metabolic drivers of PI3K activation. In vitro breast cancer models have shown that glucose restriction or pharmacological glucose transport inhibition results in decreased Akt phosphorylation, reduced mTORC1 activity, and enhanced sensitivity to endocrine agents [102]. While SGLT2 is minimally expressed in breast cancer cells themselves, systemic metabolic stress induced by SGLT2i, characterized by reduced glucose, insulin, and IGF-1 availability, may phenocopy the effects of direct PI3K/mTOR inhibition, albeit through a less toxic and more physiologic mechanism (Fig. 2) [103].

### AMPK activation, energetic stress, and reversal of adaptive survival programs

Another hallmark of endocrine resistance is the acquisition of energetic resilience, allowing tumor cells to survive in estrogen-depleted environments [104]. Activation of AMP-activated protein kinase (AMPK) represents a critical counter-regulatory mechanism to anabolic growth signaling. AMPK activation inhibits mTOR, suppresses lipid and protein synthesis, and promotes autophagy and oxidative metabolism [105]. SGLT2i have been shown, both systemically and in tumor-adjacent tissues, to activate AMPK signaling through reduced intracellular glucose flux and altered cellular energy balance [106]. Preclinical breast cancer studies demonstrate that AMPK activation enhances responsiveness to endocrine therapy by suppressing ER-independent proliferative signaling and destabilizing pro-survival metabolic programs [107]. This raises the hypothesis that SGLT2i-induced AMPK activation may function as a metabolic “brake” capable of modulating adaptive rewiring processes implicated in endocrine resistance (Table 3).

### Adipose tissue–tumor crosstalk, inflammation and leptin-driven resistance

Obesity and visceral adiposity are strongly associated with poorer outcomes and reduced endocrine therapy efficacy in HR+ breast cancer [108]. Adipose tissue is an active endocrine organ that produces adipokines, most notably leptin, which promotes ER signaling, aromatase expression, inflammation, and resistance to endocrine therapy [109]. Leptin activates JAK/STAT, MAPK, and PI3K pathways, all of which contribute to estrogen-independent tumor growth. SGLT2i reduce visceral adiposity and circulating leptin levels while increasing adiponectin, an adipokine with anti-proliferative and insulin-sensitizing properties (Fig. 2) [110]. By remodeling the adipokine milieu, SGLT2i may attenuate adipose-driven endocrine resistance mechanisms, particularly in postmenopausal women receiving AIs, where peripheral estrogen synthesis and adipose inflammation play a central role (Fig. 2) [111].

Chronic low-grade inflammation is increasingly recognized as a driver of endocrine resistance. Pro-inflammatory cytokines such as IL-6 and TNF-α activate STAT3 and NF-κB signaling, which can suppress ER expression, promote epithelial-to-mesenchymal transition (EMT), and induce stem-like phenotypes associated with therapy resistance [112]. SGLT2i consistently reduce systemic inflammatory mediators and oxidative stress markers. As summarized in Fig. 2, inflammatory cytokine networks involving IL-6/STAT3, TNF-α/NF-κB, and IL-17 signaling may simultaneously contribute to endocrine-resistant tumor phenotypes and maladaptive cardiovascular remodeling, providing a biologically plausible framework through which systemic metabolic interventions could exert dual cardiometabolic effects. In preclinical cancer models, reduced inflammatory signaling has been linked to restoration of ER expression and increased sensitivity to endocrine agents [113, 114] (Table 3). Although direct evidence in breast cancer patients is currently lacking, the anti-inflammatory effects of SGLT2i provide a biologically plausible mechanism by which these agents could stabilize ER-dependent phenotypes and delay the emergence of resistant clones (Fig. 2).

### Tumor microenvironment and metabolic competition

Endocrine-resistant tumors often display increased reliance on aerobic glycolysis and altered nutrient competition within the tumor microenvironment [115]. By lowering systemic glucose availability and increasing circulating ketone bodies, SGLT2i may create a metabolically unfavorable environment for highly glycolytic, endocrine-resistant cancer cells [116]. Experimental data suggest that ER-positive breast cancer cells are less metabolically flexible than triple-negative counterparts and may be particularly vulnerable to systemic metabolic stress [117]. This concept aligns with emerging models in which metabolic therapies act not as direct cytotoxics, but as selective pressure modifiers, reshaping tumor evolution and delaying resistance rather than inducing immediate tumor regression [118]. Figure 2 conceptually integrates these interactions by positioning SGLT2 inhibition as a systemic metabolic intervention capable of modulating interconnected inflammatory, energetic, and insulin-dependent pathways across tumor, adipose, and cardiovascular compartments. However, this conceptual framework remains largely speculative in HR+ breast cancer, as no dedicated preclinical or clinical studies have yet demonstrated that SGLT2 inhibition directly alters the timing, molecular evolution, or therapeutic responsiveness of endocrine-resistant disease. Nevertheless, emerging translational evidence suggests that SGLT2 inhibition may modulate cardiometabolic stress pathways relevant to endocrine-resistant settings. In a recent experimental study using hyperglycemic hiPSC-derived cardiomyocytes exposed to the PI3Kα inhibitor alpelisib and fulvestrant, dapagliflozin attenuated mitochondrial dysfunction, oxidative stress, inflammasome activation, and inflammatory signaling, supporting a potential role for SGLT2i in mitigating the cardiometabolic toxicity associated with PI3K-targeted endocrine therapy combinations [119]. Although these findings do not demonstrate modulation of endocrine sensitivity per se, they provide mechanistic support for further investigation of SGLT2 inhibition within metabolically vulnerable endocrine-resistant disease conditions.

Resistance to endocrine therapy remains a central clinical challenge in hormone receptor–positive breast cancer and a major driver of late recurrence and disease progression [120]. Increasing evidence indicates that endocrine resistance is not solely a consequence of estrogen receptor alterations but rather the result of adaptive metabolic rewiring, activation of compensatory growth pathways, and inflammatory remodeling of the tumor microenvironment [120]. Within this framework, SGLT2i have emerged as biologically plausible modulators of several interconnected axes implicated in endocrine escape [121]. By attenuating hyperinsulinemia, suppressing insulin–IGF-1–driven PI3K–Akt–mTOR signaling, activating AMPK-dependent energy stress responses, and remodeling adipokine and inflammatory signaling, SGLT2i may indirectly stabilize estrogen-dependent phenotypes and delay the emergence of endocrine-resistant clones (Fig. 2) [122, 123]. Although direct clinical evidence remains limited, the convergence of metabolic, inflammatory, and signaling pathways targeted by SGLT2 inhibition provides a biologically coherent rationale for future investigation of these agents as potential adjunctive metabolic modulators in endocrine-resistant disease, particularly in metabolically vulnerable patients receiving long-term estrogen-depleting therapies [124] (Table 3). Future studies integrating metabolic phenotyping, longitudinal endocrine response assessment, ESR1 mutational dynamics, and adipose–tumor interaction models will be necessary to determine whether SGLT2i can meaningfully influence endocrine resistance biology.

## Safety, oncologic neutrality, and translational gaps

The available evidence indicates that the safety profile of SGLT2i in oncology settings is broadly consistent with that observed in the general cardiometabolic population, although cancer patients present unique context-dependent vulnerabilities that require careful clinical consideration. During active anticancer therapy, factors such as gastrointestinal toxicity, reduced oral intake, cachexia, and concomitant diuretic or renin–angiotensin–aldosterone system blockade may increase susceptibility to volume depletion and symptomatic hypotension, while immunosuppression may modestly heighten the risk of genitourinary mycotic infections [125]. Euglycemic ketoacidosis remains a rare but clinically relevant complication, particularly in settings characterized by prolonged fasting, acute illness, perioperative stress, or severe catabolic states, all of which are common along the cancer care continuum [126]. Nonetheless, with appropriate patient selection and monitoring, current data support an acceptable cardiometabolic safety profile for SGLT2i in oncology populations. From an oncologic perspective, available meta-analyses and real-world observational studies do not demonstrate an increased risk of cancer incidence, recurrence, or progression associated with SGLT2 inhibitor exposure, although oncologic endpoints are often secondary, incompletely adjudicated, and limited by follow-up duration. Preclinical signals suggesting indirect anticancer or endocrine-sensitizing effects, mediated through suppression of insulin–IGF-1–driven PI3K–Akt–mTOR signaling, activation of AMPK-dependent energy stress responses, remodeling of adipokine and inflammatory networks, and induction of metabolic stress within the tumor microenvironment, remain hypothesis-generating and require prospective validation (Fig. 2). Despite increasingly consistent associations with reduced heart failure risk and attenuation of cancer therapy–related cardiac dysfunction, the current evidence base is dominated by nonrandomized designs and is therefore vulnerable to residual confounding, endpoint heterogeneity, limited oncologic granularity, and time-dependent biases. Moreover, direct clinical evidence linking SGLT2 inhibitor use to delayed endocrine resistance in hormone receptor–positive breast cancer remains sparse and largely indirect, with no prospective trials yet incorporating resistance-specific endpoints such as duration of response to aromatase inhibitors, emergence of ESR1 mutations, or need for therapeutic escalation, all of which are increasingly recognized as clinically relevant markers of acquired endocrine resistance [127]. Therefore, the initiation of randomized trials like PROTECT, evaluating dapagliflozin for the prevention of cardiotoxicity associated with anthracycline-based chemotherapy and HER2-targeted therapy in early-stage breast cancer, represents a critical transition from associative inference to causal evaluation. Future breast cancer–specific trials will need to integrate hard cardiovascular outcomes, standardized definitions of cancer therapy–related cardiac dysfunction, detailed metabolic phenotyping relevant to endocrine therapy, and rigorous oncologic safety and efficacy endpoints to define whether SGLT2i can be safely and effectively incorporated as cardiometabolic and potentially endocrine-sensitizing agents in survivorship care (Fig. 2).

## Perspectives and conclusions

In conclusion, the current clinical evidence, anchored by large real-world propensity-matched analyses demonstrating reduced CTRCD risk (HR ~ 0.76) and meta-analytic signals indicating lower incident HF/HF hospitalization in cancer populations, supports the hypothesis that SGLT2i may serve as effective cardiometabolic modulators in oncology and survivorship care. The most consistent and biologically credible benefit pertains to HF-related outcomes and CTRCD prevention, with particularly relevant signals in anthracycline-exposed cohorts, including breast cancer. Nevertheless, definitive integration into breast cancer endocrine therapy pathways requires adequately powered, prospective randomized trials with standardized cardiotoxicity phenotyping and comprehensive oncologic annotation. In conclusion, the expanding interface between cardio-oncology, metabolism, and breast cancer survivorship positions SGLT2i as potential system-level modulators rather than organ-specific agents. To translate mechanistic and real-world evidence into clinical practice, prospective randomized trials are needed to evaluate their efficacy in breast cancer patients receiving endocrine therapy, with a focus on hard cardiovascular outcomes, standardized cancer therapy–related cardiac dysfunction endpoints, and long-term follow-up. Future studies should account for the metabolic heterogeneity of breast cancer survivors, including insulin resistance, visceral adiposity, and metabolic dysfunction–associated steatotic liver disease, and explicitly assess benefit in non-diabetic populations. In parallel, the hypothesis that SGLT2i may indirectly delay endocrine therapy resistance requires dedicated investigation using resistance-specific clinical and molecular endpoints. Equally important is the systematic evaluation of oncologic safety, treatment adherence, and long-term outcomes to ensure cardiometabolic benefit without compromising cancer control. If validated, integration of SGLT2i into multidisciplinary survivorship care pathways could represent a paradigm shift toward early, preventive cardio-oncology strategies in hormone receptor–positive breast cancer.

## Data Availability

No new data were created or analyzed in this study. This narrative review is based on previously published research, which has been cited appropriately throughout the manuscript. All data supporting the findings of this review are available in the referenced articles.

## Abbreviations

AI: Aromatase inhibitor
Akt: Protein kinase B
AMPK: AMP-activated protein kinase
ARNI: Angiotensin receptor–neprilysin inhibitor
BC: Breast cancer
BMI: Body mass index
Ca^2+^: Calcium ion
CDK: Cyclin-dependent kinase
CRP: C-reactive protein
CTRCD: Cancer therapy–related cardiac dysfunction
CVD: Cardiovascular disease
DEXA: Dual-energy X-ray absorptiometry
EMT: Epithelial-to-mesenchymal transition
ER: Estrogen receptor
ERα: Estrogen receptor alpha
ESC: European Society of Cardiology
FGF21: Fibroblast growth factor 21
GLS: Global longitudinal strain
HbA1c: Glycated hemoglobin
HDL: High-density lipoprotein
HF: Heart failure
HOMA-IR: Homeostatic Model Assessment of Insulin Resistance
HR+: Hormone receptor–positive
HTN: Hypertension
ICD: International Classification of Diseases
IGF-1: Insulin-like growth factor 1
IL: Interleukin
IL-10 R: Interleukin-10 receptor
JAK: Janus kinase
MAPK: Mitogen-activated protein kinase
MASLD: Metabolic dysfunction–associated steatotic liver disease
MACE: Major adverse cardiovascular events
MRA: Mineralocorticoid receptor antagonist
mTOR: Mechanistic target of rapamycin
mTORC1: Mechanistic target of rapamycin complex 1
NAFLD: Nonalcoholic fatty liver disease
NF-κB: Nuclear factor kappa B
NT-proBNP: N-terminal pro–B-type natriuretic peptide
PGC-1α: Peroxisome proliferator–activated receptor gamma coactivator 1-alpha
PI3K: Phosphoinositide 3-kinase
RAAS: Renin–angiotensin–aldosterone system
ROS: Reactive oxygen species
RCT: Randomized controlled trial
RR: Relative risk
RYR2: Ryanodine receptor 2
SERCA2a: Sarcoplasmic/endoplasmic reticulum Ca^2 +^ -ATPase 2a
SERM: Selective estrogen receptor modulator
SERD: Selective estrogen receptor degrader
SGLT2: Sodium–glucose cotransporter 2
SGLT2i: Sodium–glucose cotransporter 2 inhibitor
STAT3: Signal transducer and activator of transcription 3
T2DM: Type 2 diabetes mellitus
TGF-β: Transforming growth factor beta
TNF-α: Tumor necrosis factor alpha
